# The Development of a Multilayer Transdermal Patch Platform Based on Electrospun Nanofibers for the Delivery of Caffeine

**DOI:** 10.3390/pharmaceutics17070921

**Published:** 2025-07-16

**Authors:** Jorge Teno, Zoran Evtoski, Cristina Prieto, Jose M. Lagaron

**Affiliations:** 1 R&D Department, Bionanopharma S.L., Calle Algepser 65 nave 3, 46980 Paterna, Valencia, Spain; jteno@bionanopharma.com; 2 Novel Materials and Nanotechnology Group, Institute of Agrochemistry and Food Technology (IATA), Spanish Council for Scientific Research (CSIC), Calle Catedrático Agustín Escardino Benlloch 7, 46980 Paterna, Valencia, Spain; zoran.evtoski@iata.csic.es (Z.E.); cprieto@iata.csic.es (C.P.)

**Keywords:** electrospinning, transdermal delivery

## Abstract

**Background/Objectives**: The work presented herein focused on the development and characterization of a transdermal caffeine platform fabricated from ultrathin micro- and submicron fibers produced via electrospinning. **Methods**: The formulations incorporated caffeine encapsulated in a polyethylene oxide (PEO) matrix, combined with various permeation enhancers. A backing layer made of annealed electrospun polycaprolactone (PCL) facilitated the lamination of the two layers to form the final multilayer patch. Comprehensive characterization was conducted, utilizing scanning electron microscopy (SEM) to assess the fiber morphology, attenuated total reflectance Fourier transform infrared spectroscopy (ATR-FTIR) for chemical detection and to assess the stability of the caffeine, and differential scanning calorimetry (DSC) along with wide-angle X-ray scattering (WAXS) to analyze the physical state of the caffeine within the fibers of the active layer. Additionally, Franz cell permeation studies were performed using both synthetic membranes (Strat-M) and ex vivo human stratum corneum (SC) to evaluate and model the permeation kinetics. **Results:** These experiments demonstrated the significant role of enhancers in modulating the caffeine permeation rates provided by the patch, achieving permeation rates of up to 0.73 mg/cm^2^ within 24 h. **Conclusions:** This work highlights the potential of using electro-hydrodynamic processing technology to develop innovative transdermal delivery systems for drugs, offering a promising strategy for enhancing efficacy and innovative therapeutic direct plasma administration.

## 1. Introduction

Transdermal drug delivery systems (TDDSs), commonly referred to as “patches”, are an alternative to oral drug administration and hypodermic injections. TDDSs offer several advantages over other administration routes, including bypassing first-pass metabolism, their ease of self-administration, a reduced dosing frequency, and minimized gastrointestinal side effects. Despite these benefits, developing the ability to deliver most drug molecules via the transdermal route remains one of the greatest challenges in TDDS development. The primary barrier to transdermal drug delivery (TDD) is the stratum corneum (SC), the outermost layer of the skin. The SC has a dense cellular architecture that significantly limits percutaneous absorption. Composed of non-polar lipids and neutral keratin proteins, the SC requires drug molecules to have adequate solubility in both water and oil to permeate through the skin effectively [1,2]. Drug molecules can penetrate the skin via three main pathways: (i) intracellular diffusion across SC corneocytes, (ii) penetration through the SC’s intercellular lipid spaces, and (iii) penetration through skin appendages. Among these, the intercellular lipid domain of the SC is considered the principal pathway for drug penetration by the scientific community [3,4].

Chemical strategies to reduce the effect of the barrier include the use of penetration enhancers. Transdermal enhancers are introduced into patches to overcome the excellent barrier function of the SC by temporarily weakening the skin barrier. They are effective in increasing skin permeation rates and improving the skin delivery of topically active drugs, reducing the permeability barrier without causing significant damage to cells. Enhancers can also modify the properties of the formulation to promote drug delivery [5,6,7]. They can be classified according to their chemical composition into different groups, such as alcohols, fatty acids, glycols, and surfactants, among others [8]. Depending on their chemical properties and the interactions between the drugs and enhancer, the choice of an appropriate enhancer should anticipate the mechanism of action of the drug and improve the drug’s permeation. A wide range of enhancers shows potent permeation-enhancing effects; nevertheless they are drug-specific, and they do not have the same effect with all drugs. For example, Span 80 has been reported to increase the permeation of diclofenac and olanzapine by 4.4 and 1.6 folds, respectively, but it was seen to reduce the permeation of flurbiprofen by only 0.8 folds [9,10]. Xu et al. studied the specificity of the enhancement effects of polyglyceryl-3-dioleate (POCC) on ten drugs. They found that drugs with a low polar surface area value exhibited high dispersion after the addition of POCC. Furthermore, the permeation of drugs with a low polar surface area and polarizability was enhanced more significantly by POCC, which was illustrated by the interactions among the drugs, POCC, and skin lipids [11].

Drug-in-adhesive patches are the most commonly used system for combining drugs and enhancers in an adhesive matrix that remains in direct contact with the skin. However, these systems often face challenges, such as limited drug loading, relatively low permeation, limited drug choices or compatibility issues, skin irritation, etc. [12]. Addressing some or all of these disadvantages often involves exploring alternative technologies, such as multilayer systems [13,14], microneedles [15,16], or electrospun nanofibers, to enhance the drug delivery efficiency and expand the range of deliverable therapeutics [17,18].

In recent years, the electrospinning technique has gained attention as a promising method to increase the bioavailability of active ingredients. Electrospinning is one of the simplest and most versatile methods for producing ultrathin fibers, so-called electrospun nanofibers, with applications across a broad range of materials. Notably, the rapid solidification of electrospun nanofibers and the interactions between drugs and fiber matrices have been shown to improve drug dispersion [18,19]. This technique allows for the use of a wide variety of polymers, making it adaptable for diverse drug formulations and therapeutic applications. Moreover, the high surface area of electrospun fibers enhances drug dispersion within the patch, potentially enabling more consistent and controlled drug delivery. Electrospinning also offers the potential to incorporate drugs with poor solubility into nanofibers, thereby improving their solubility and bioavailability. Additionally, this technique accommodates a wide range of drug types, including small molecules, peptides, and proteins, making electrospun fibers a versatile platform for drug encapsulation, and can protect drugs from degradation or premature release, increasing their stability in the delivery system [20,21,22].

Caffeine, which has the chemical compound name 1,3,7-trimethylxanthine, is being used in some applications. Caffeine is utilized in the pharmaceutical industry for various purposes. Some of the most common applications of caffeine in medications include its use as an adjunct in analgesics to enhance their efficacy [23], as a central nervous system (CNS) stimulant [24], in dermatological applications [25], etc. Caffeine, applied topically, is available in the form of creams and lotions, primarily because of its diverse effects on the skin [26]. It is also recognized for its potential to slow down the skin aging process by offering protection against ultraviolet light, absorbing UV radiation, and reducing the risk of skin cancer. Additionally, caffeine is an active ingredient in anti-cellulite formulations, known for its robust antioxidant properties. It can enhance blood microcirculation within the skin and promote hair growth by inhibiting the activity of the enzyme 5-α-reductase [27]. Caffeine, being considered a rather hydrophilic drug with a log P of −0.07, has also been used as a model drug for active principle ingredients with low skin permeability and low water solubility [28]. The logarithm of the partition coefficient (log P), which is defined as the ratio between a compound’s concentrations in two immiscible solvents at equilibrium, typically n-octanol (representing the lipid phase) and water (representing the aqueous phase). A positive log P value indicates greater solubility in the lipid phase, suggesting that the compound is lipophilic, while a negative log P value reflects higher solubility in the aqueous phase, indicating that the compound is hydrophilic. Caffeine, together with other actives, has previously been encapsulated in monolayers to be used as potential oral dispersible films with other electrospun fiber materials such as polyvinyl alcohol (PVOH) and polyvinylpyrrolidone (PVP) [28,29].

The aim of this seminal study was to develop a multilayer electrospun patch platform from pharma-grade polymeric materials to evaluate the transdermal drug delivery of caffeine. Hydrophilic polyethylene oxide (PEO) was used as a model polymer to provide skin adhesion and homogeneous drug delivery, and the biopolymer polycaprolactone (PCL) was selected as the hydrophobic backing layer. The electrospun nanofibers were characterized using scanning electron microscopy (SEM), attenuated total reflectance Fourier transform infrared spectroscopy (ATR-FTIR), and wide-angle X-ray scattering (WAXS). The drug permeation was analyzed using a Franz diffusion cell tool, initially with a multilayer synthetic membrane, followed by testing on human biopsy stratum corneum membranes to assess the ex vivo permeation and permeation kinetics of the optimal candidate.

## 2. Materials and Methods

### 2.1. Materials

Caffeine (powder, Reagent Plus^®^), polyethylene glycol (PEG, MW400), oleic acid (OA, technical grade, 90%), and chloroform (ACS reagent, ≥ 99.8%) were purchased from Sigma-Aldrich (Madrid, Spain). Methanol (MeOH) and propylene glycol (PG, pharma-grade) were supplied by Panreac Química S.L.U. (Castellar del Vallès, Barcelona, Spain). Polyethylene oxide (PEO, Mw 200,000 Da) was purchased from the Dow Chemical Company (Montgomeryville, PA, USA), and poly-ε-caprolactone (PCL, Resomer C212) was supplied by Evonik Industries (Essen, Germany), while Eucalyptus Globulus Leaf Oil (EUC, 80–85% of 1,8-cineole) was supplied by Gran Velada (Aragon, Spain). Dulbecco’s phosphate-buffered saline (DPBS) solution without calcium and magnesium ions was purchased from Thermo Fisher Scientific (Waltham, MA, USA). All the polymers and reagents were used as received without further purification.

### 2.2. Solution Preparation

The solutions for use in the active adhesive layer were prepared by first dissolving the polymer in the appropriate solvent. Caffeine and the corresponding enhancers were then added to the solution under continuous stirring at room temperature. Caffeine is known to be soluble in a chloroform/methanol solvent mixture. In all cases, with and without enhancers, stable and transparent solutions were obtained, with no precipitation observed in the injector during and after the electrospinning process. All the solutions were prepared using a PEO concentration of 9% wt. The developed solutions are outlined in Table 1.

### 2.3. Electrospinning

The electrospinning process was carried out in a Fluidnatek^TM^ LE-100 equipped with a 5-multi-emitter injector system from Bioinicia S.L. (Valencia, Spain) and an air-conditioned unit. Table 2 shows the electrospinning parameters used for each solution. The environmental conditions were kept at 30 °C and 25% relative humidity (RH) for all the electrospinning processes.

**Figure 1:**
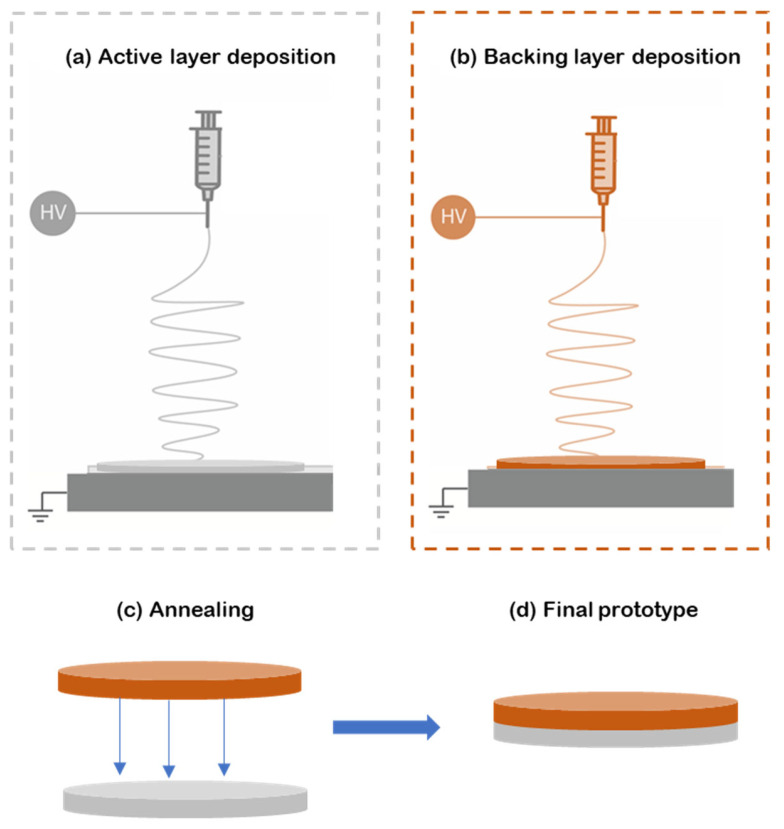
Diagram of the formulation of the multilayer patch. (**a**) Electrospinning of the active PEO-based layer (gray); (**b**) electrospun PCL backing layer produced separately from the previous layers (orange); (**c**) lamination at a low temperature by annealing the backing layer to the active layer; (**d**) final multilayer patch prototype. In this scheme HV means High Voltage.

For this study, bilayer patches were prepared as follows. First, the reservoir layer was made from electrospun PEO fibers containing caffeine and the correspondent enhancers (Figure 1a). Then, the hydrophobic backing layer (BL) made of electrospun PCL fibers was electrospun separately (Figure 1b) and laminated to the previously prepared material using a hot press (Carver 4122, Wabash, IN, USA) for 20 s without applying pressure to assemble the whole multilayer patch (Figure 1c,d). The surface density, a way to measure the sample thickness in non-woven mats, was optimized at 10 g/m^2^ for the PCL backing layer and 70 g/m^2^ for the active PEO-based layer.

### 2.4. Human Skin Sample Preparation

Skin biopsy specimens from two healthy volunteers, a 39-year-old man with white skin and some thick hair follicles and a 68-year-old woman with white skin, stored at −80 °C, were provided by the Biobank of the Valencia Region, Spain. Briefly, the full-thickness skin was sealed in sterile plastic bags and frozen at −80 °C within 24 h of removal to be used within a month. Before preparation, the skin was thawed to room temperature, and any excess fat was carefully removed. The subcutaneous tissue was meticulously excised using an aseptic scalpel, and the skin was cut into pieces according to the requirements of the permeation experiments. The skin pieces were immersed in preheated Dulbecco’s phosphate-buffered saline (DPBS) at 60 °C for 45–60 s. The excess DPBS was removed, and the stratum corneum layer was gently peeled away from the biopsy using sterile forceps. The SC is the outermost hydrophobic barrier layer in the skin.

### 2.5. Characterization

#### 2.5.1. Fiber Morphology (SEM)

The fiber morphology of the different patches produced was analyzed by scanning electron microscopy (SEM) using a Phenom XL G2 Desktop microscope (Thermo Fisher Scientific, Waltham, MA, USA) with an electron beam acceleration of 5 kV. The average fiber diameter based on at least 100 fibers was determined using Phenom ProSuite Software (https://www.phenom-world.com (accessed on 4 January 2024)) to analyze the SEM images.

#### 2.5.2. Assessment of In Vitro Release of Caffeine Patches Using Franz Diffusion Cell

The release experiments were performed using Franz diffusion cell equipment at 35 °C with an effective diffusion area of 1 cm^2^. In the first experiment, a polyether sulfone membrane, Strat-M^®^, was placed between the donor and receptor chambers of the Franz diffusion cell. The permeation experiments with this synthetic membrane were performed in triplicate. To maintain the temperature of the receptor solution at 35 °C, a water jacket was connected to a water bath with continuously recirculating water. The patches were cut into pieces with a 1 cm^2^ effective surface and placed on top of the Strat-M membrane; then the assembly was mounted on the receptor chamber containing 8.0 mL of a PBS buffer solution (pH 7.4). The upper and lower parts of the Franz cell were sealed with parafilm and fastened together by means of a clamp. The in vitro caffeine permeation was monitored for different lengths of time: 1, 3, 6, 8 and 24 h. A certain amount of the solution, 0.5 mL, was withdrawn from the receptor chamber and then replaced with the same volume of the PBS solution (pH 7.4) to keep the sinking volume constant throughout the analysis. The caffeine concentration was determined using an HPLC Acquity^®^ TQD (Waters, Milford, MA, USA) equipped with a C18 column (BEH, 1.7 µm, Waters) and a mass detector.

The patch displaying the best caffeine permeation results obtained using Strat-M was further evaluated using two ex vivo human skin specimens from the two donors. Each specimen was mounted onto the Franz diffusion cell with the epidermis facing downward and the stratum corneum side in contact with the patch under investigation.

#### 2.5.3. Fourier Transform Infrared (FTIR) Spectroscopy

The presence of caffeine in the polymer matrix was evaluated using Fourier-transformed infrared spectroscopy and measured with a Bruker Tensor 37 FT-IR Spectrometer (Bruker, Ettlingen, Germany) paired with a Golden Gate attenuated total reflectance (ATR) sampling accessory (Specac Ltd., Orpington, UK). Spectra were obtained from the average of 64 scans in the range of 4000–600 cm^−1^, with a resolution of 4 cm^−1^.

#### 2.5.4. Differential Scanning Calorimetry (DSC)

The electrospun fibers were studied by differential scanning calorimetry (DSC) on a DSC-8000 analyzer equipped with an Intracooler 2 cooling accessory from PerkinElmer, Inc. (Waltham, MA, USA). Approximately 3 mg of each sample was placed in a standard aluminum pan and heated from 0 to 300 °C at a rate of 10 °C/min, using a nitrogen flow of 20 mL/min as the sweeping gas.

#### 2.5.5. Wide-Angle X-Ray Scattering (WAXS)

The samples were analyzed using a Bruker AXS D4 Endeavor diffractometer (Bruker, Ettlingen, Germany). The samples were scanned by wide-angle X-ray scattering at room temperature in the reflection mode, using incident Cu K-alpha radiation (Cu Kα = 1.54 Å), while the generator was set at 40 kV and 40 mA. The data were collected over a range of scattering angles (2θ) in the 5–40° range. To estimate the average crystallite size of caffeine, the Scherrer equation was used:

(1) $$\;D=\frac{K\;\lambda }{\beta \;\cos\theta }$$

where *D* is the crystallite size, *K* is the shape factor, *λ* is the XRD radiation wavelength, and *β* is the full width at half maximum of the peak in radians.

#### 2.5.6. Kinetic Modeling of Ex Vivo Human Skin Permeation

To analyze the kinetics of the in vitro drug release behavior of the patch, semi-empirical mathematical models were fitted to the curve of caffeine permeation through human skin. The Korsmeyer–Peppas model was used to fit the experimental data since no burst release was observed that would have justified the use of other, modified models such as the Ritger–Peppas model.

The Korsmeyer–Peppas model is mathematically described as follows:

(2) $$Q=Kt^{n}$$

where Q is the amount of the drug released over time t, K is the constant of incorporation of the structural modifications and geometrical characteristics of the system (also considered the release velocity constant), and n is the release exponent (which depends on the drug type’s polydispersity, geometry, and transport) as a function of time t. Depending on the release exponent, the diffusional release mechanisms were classified as pseudo-Fickian diffusional behavior if n < 0.5, Fickian diffusion if n = 0.5, non-Fickian diffusion if 0.5 < n < 1, case II transport (zero-order release) if n = 1, and super case II transport if n > 1 [30].

## 3. Results and Discussion

### 3.1. The Fiber Morphology (SEM) of the Active Layer

**Figure 2:**
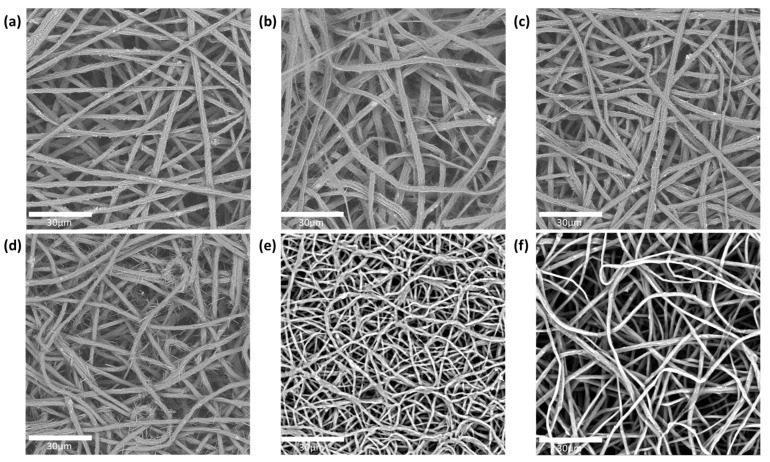
SEM images of PEO/caffeine active layers containing various enhancers: (**a**) without enhancer; (**b**) oleic acid; (**c**) polyethylene glycol; (**d**) propylene glycol and oleic acid; (**e**) polyethylene glycol and oleic acid; and (**f**) polyethylene glycol and eucalyptol.

The fiber morphology of the different PEO/caffeine patches was analyzed by SEM. On the one hand, the fibers without an enhancer or containing one enhancer in their formulation displayed similar morphologies. These fibers displayed a flattened morphology (see Figure 2). On the other hand, the fibers containing two enhancers displayed a rounded morphology. This difference could be attributed to the solvent evaporation rate during the electrospinning process; when the solvent evaporated more rapidly, the fibers collapsed, leading to the development of a planar morphology. If the solvent evaporated more slowly, the fibers could maintain a round morphology. It is interesting to note that some needle-like caffeine crystals were observed protruding from the fiber surface in some of the samples, with the exception of the samples (Figure 2e,f), containing polyethylene glycol and oleic acid and polyethylene glycol and eucalyptol mixtures, respectively, which did not show this feature [31]. The fiber sizes corresponding to the various active layers with different enhancers are summarized in Table 3. The fibers containing PEG and OA as enhancers displayed a smaller diameter than the rest of the fibers.

### 3.2. The Effect of the Enhancer on the Permeation of Caffeine

**Figure 3:**
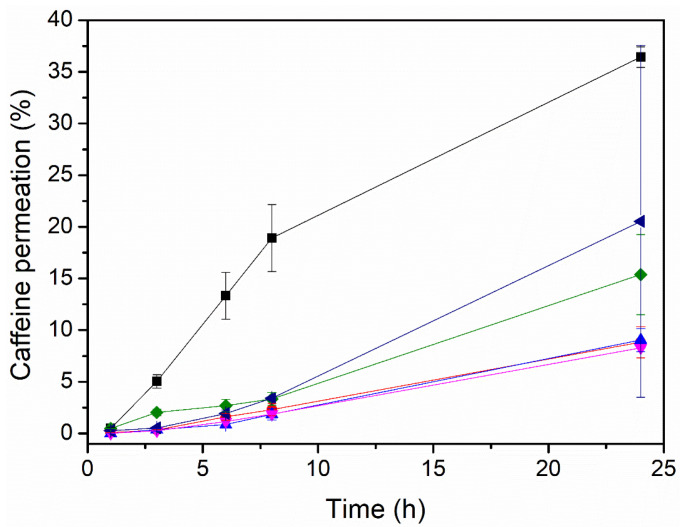
Caffeine permeation in µg/cm^2^ of the multilayer PCL + PEO/caffeine patches with different enhancers in the PEO active layer: 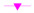 without an enhancer, 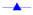 oleic acid, 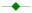 polyethylene glycol, 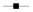 polyethylene glycol and oleic acid, 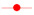 propylene glycol and oleic acid, and 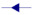 polyethylene glycol and eucalyptol oil.

Figure 3 and Table 4 display the data on the permeation through the Strat-M membrane for caffeine-loaded electrospun PEO patches with different enhancers. The variations in the initial caffeine content per cm^2^, theoretically calculated, were the result of the different grammages generated during the production process for each sample, with the ratio of excipients/caffeine always being 80/20, as shown in Table 1. As can be observed, the enhancer had a strong influence on the caffeine permeation. The patches containing PEG_OA, PEG_EUC, and PEG displayed higher caffeine permeability than the patches containing the other enhancers. The compatibility of the enhancers with caffeine, from the most soluble to the least soluble, was as follows: PEG, the PEG_OA mixture, the PEG_EUC mixture, PG, and the PG_OA mixture and OA. Interestingly, the patch containing the PEG_OA enhancer mixture displayed the highest caffeine permeability over 24 h, around 36 ± 1%. This result is in good agreement with observations ranking this mixture as being highly compatible with caffeine and the fibers not showing detectable traces of caffeine crystals at their surface. In addition, PEG is considered a non-ionic surfactant as it can potentially interact with biological membranes, especially skin, causing an increase in their permeability and the transmembrane transport of solutes, which, together with the effects of OA, could synergistically reduce the diffusional resistance of the skin by interacting with the lipid matrix, since OA has been reported to increase lipids’ fluidity [8,32]. In view of these results, the PEO_CAF_PEG_OA patch was selected for an additional test of the permeation through human SC biopsies.

### 3.3. Fourier Transform Infrared (FTIR) Spectroscopy

**Figure 4:**
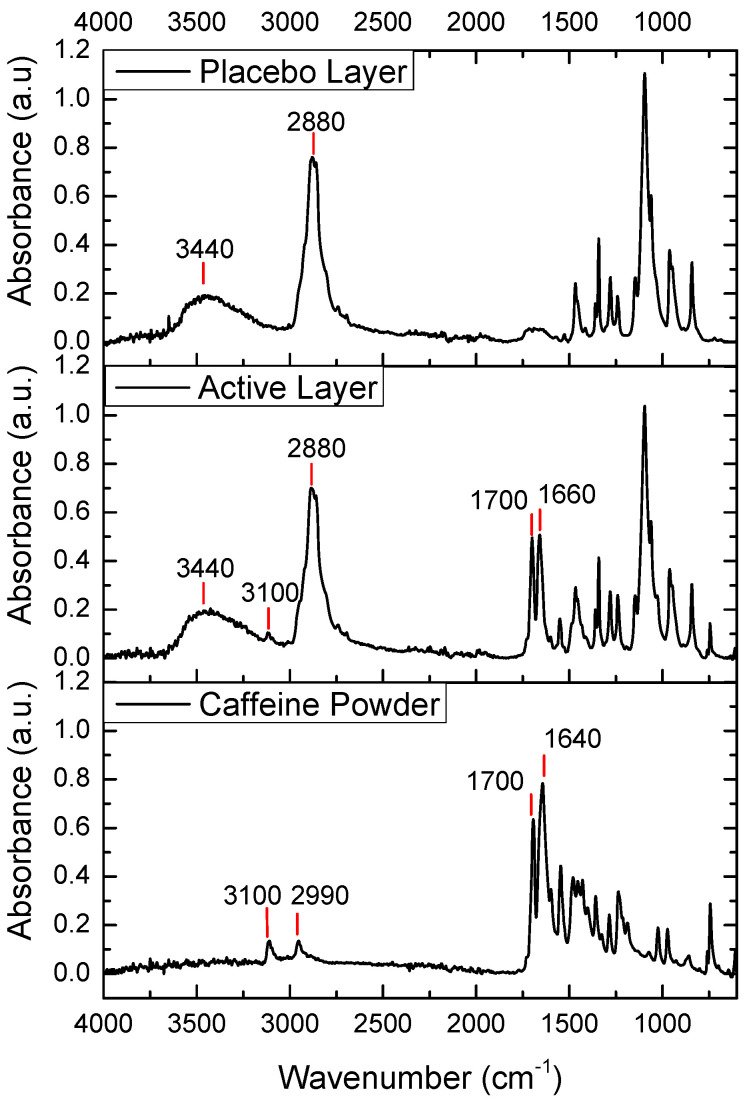
FTIR spectra of the caffeine powder, the active monolayer (PEO_CAF_PEG_OA), and the placebo monolayer (PEO_PEG_OA).

The FTIR spectra of pure caffeine, the placebo layer, and the active layer are shown in Figure 4. The FTIR spectrum of caffeine powder showed two peaks at 3110 cm^−1^ and 2955 cm^−1^ corresponding to aromatic C-H stretching vibrations. The peak at 1643 cm^−1^ was due to -C=N ring stretching, and the peak at 1700 cm^−1^ was attributed to C-O stretching vibration in the cyclic hydrocarbons [33]. In the spectrum of the placebo layer, we observed two strong peaks at 2880 cm^−1^ and 3440 cm^−1^, corresponding to C-H bond stretching and O-H stretching vibration, respectively. In the case of the spectrum of the caffeine-containing layer, the main peaks of the placebo-layer components could also be observed, a broad peak at 3440 cm^−1^ and a peak at 2800 cm^−1^. Interestingly, the main caffeine peaks mentioned above were also seen, but with the caffeine band exhibiting a stronger relative intensity and shift towards higher wavenumbers at 1640 cm^−1^. The latter could have been associated with some potential interactions between the caffeine and the excipients in the active layer.

### 3.4. Differential Scanning Calorimetry (DSC)

**Figure 5:**
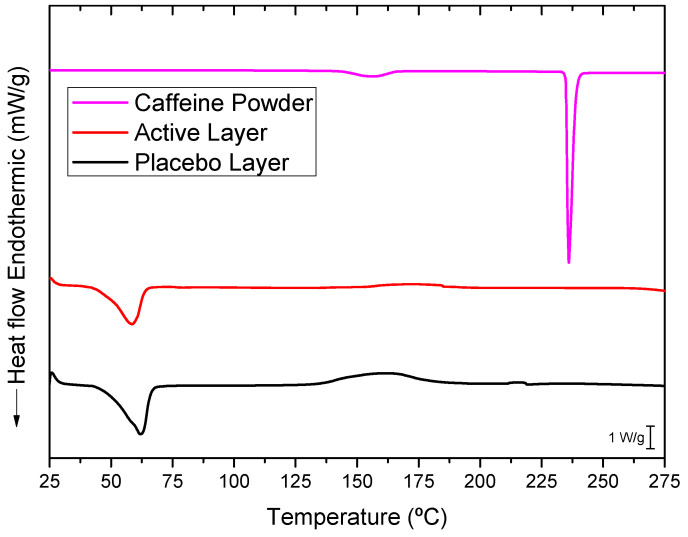
Typical DSC thermograms of the caffeine powder, the active monolayer (PEO_CAF_PEG_OA), and the placebo monolayer (PEO_PEG_OA).

The selected PEO_CAF_PEG_OA active layer and its placebo counterpart were further analyzed to elucidate the physical states of caffeine and the polymer matrix. Figure 5 presents the DSC thermograms of the active layer, the placebo layer (without caffeine), and pure caffeine powder. The thermogram of pure, commercial, as-received caffeine exhibited a sharp and intense endothermic peak at around 236 °C, corresponding to the melting of caffeine crystals. The electrospun PEO_PEG_OA placebo layer displayed a distinct endothermic peak at 61.96 °C with an enthalpy of fusion of 211.43 J/g (normalized to the mass of the PEO in this layer), associated with the melting characteristics of the PEO crystals. Similarly, the caffeine-loaded fibers showed a PEO melting point at 58.54 °C with an enthalpy of fusion of 205 J/g (normalized to the mass of PEO in this layer). A small decrease in the melting point could have been associated with the presence of caffeine; this does not, however, seem to impair the PEO polymer’s crystallization significantly. Interestingly, the melting peak of caffeine was absent in the thermogram of the caffeine-loaded layer. This absence may be attributed to the electrospinning process inducing a high degree of caffeine amorphization within the fibers that did not contribute to a coherent, measurable calorimetric signal. Alternatively, since the melting point of caffeine is much higher than that of PEO, it is possible that any potential caffeine crystals could have dissolved in the molten PEO during the DSC run. Thus, to further investigate the physical state of the drug, WAXS experiments were also conducted on the active layer [29].

### 3.5. Wide-Angle X-Ray Scattering (WAXS)

**Figure 6:**
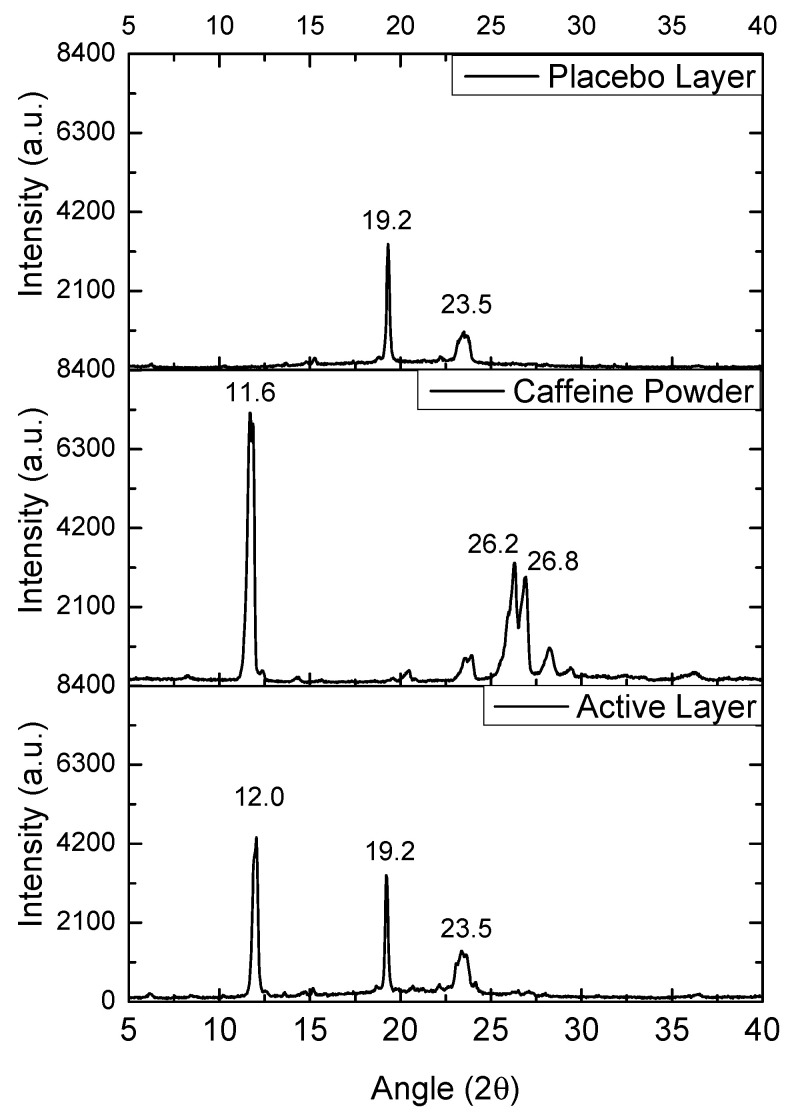
WAXS spectra of the caffeine powder, the PEO_CAF_PEG_OA active monolayer, and the PEO_PEG_OA placebo monolayer.

The WAXS patterns of the commercial caffeine powder, the selected PEO_CAF_PEG_OA active layer, and the PEO_PEG_OA fiber placebo layer were analyzed to assess the potential crystalline structure of the materials. Figure 6 shows the diffractograms of the mentioned samples. The placebo layer showed two main peaks at 19.2° and 23.5°, attributed to the orthorhombic phase of PEO. The caffeine powder diffractogram showed several sharp peaks, attributed to the crystalline nature of the supplied drug. The most intense diffraction peak was observed at 11.6°, which was attributed to the (001) crystalline plane, and two other intense crystalline peaks were present at 26.2° and 26.8°, which were clearly ascribable to the most thermodynamically stable polymorph of caffeine, i.e., the β form [34]. In the case of the PEO_CAF_PEG_OA active layer, the peaks corresponding to the orthorhombic phase of PEO were clearly present at the same diffraction angles (19.2° and 23.5°). Interestingly, another peak was observed at 12.0°, which could only be ascribed to caffeine. This observation suggests that although no endothermic signal was observed by DSC, some crystals of caffeine seem to have been created within the fibers. By applying the Scherrer Equation (1) to the only diffraction peak of caffeine, seen at ca. 11.6°, the estimated average crystal size for the as-received powder was around 20 nm, whereas for the active layer it was around 25 nm. So, it appears that within the fibers of the active layer, there existed some very small crystals of caffeine that were highly dispersed and distributed along the fiber mat, which were not detected, as in other compositions of the active layer, by SEM when analyzing the fiber morphology, nor by DSC. The formation of crystals of caffeine during the electrospinning process is rather unusual, as the process typically produces amorphous forms of the API, as previously reported in polyvinyl alcohol (PVOH) and polyvinylpyrrolidone (PVP) [28,29].

### 3.6. Ex Vivo Human SC Permeation and Modeling of Caffeine

**Figure 7:**
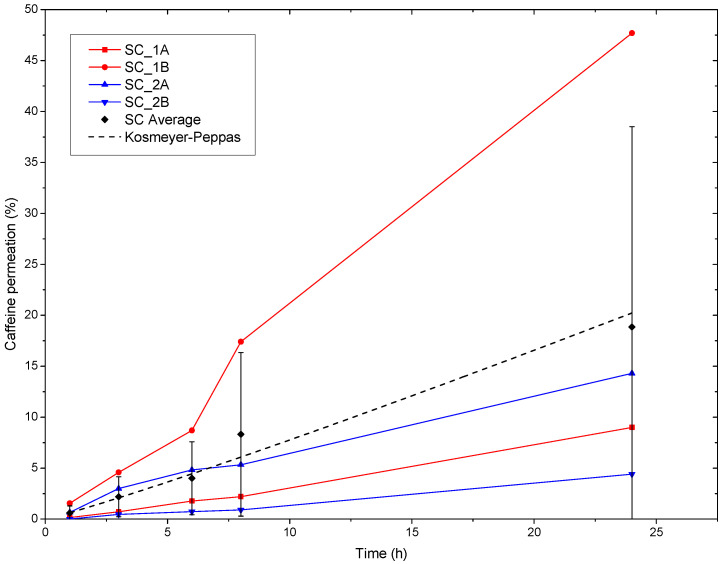
Caffeine permeation through ex vivo human SC specimens of the PCL + PEO_CAF_PEG_OA multilayer patch. SC-1A and B correspond to the SC specimens of the 39-year-old man, and SC-2A and B correspond to the SC specimens of the 68-year-old woman. The SC Average corresponds to the average of all the specimens, and the dotted line corresponds to the fit to the Kosmeyer–Peppas model.

The ex vivo human SC permeation curves can be seen in Figure 7. In this study, we observed that the variation in the permeation between the specimens was higher than that with the synthetic membrane, especially over longer testing times, most likely due to the small size of the SC tested in the Franz cell equipment, i.e., 1 cm^2^. Small skin samples could offer statistically relevant morphological features that are different between donors, such as a variable density of thick hair follicles. Additionally, SC specimens may also undergo variable aging with an increasing testing time. In addition, Figure 7 shows that the SC exhibited a different average permeation profile compared to the synthetic membrane, with the latter showing more bursts over shorter times. The average permeated caffeine value after 24 h was around 19%, i.e., 0.38 mg/cm^2^, of the initial caffeine content in the patch if all four specimens, two from each of the two donors, are considered. Figure 7 also shows that one specimen from the male donor exhibited comparatively high permeability, while for the other three, the caffeine permeation was lower. Furthermore, Eman et al. reported average caffeine permeability fluxes through human SC for high to very high concentrations of caffeine solutions, i.e., 31.3 mg/mL to 81.5 mg/mL, corresponding to 2.2 μg/cm^2^ h and 25.6 μg/cm^2^ h, respectively [35]. From this study, permeations of 0.05 mg/cm^2^ and 0.61 mg/cm^2^ could be inferred after 24 h. The authors reported significant increases not only in the permeation but also in the variability among the specimens when using enhancers such as OA.

The model of the kinetics of permeation, shown in Figure 7, was fitted with the Korsmeyer-Peppas model (Equation (2)), which provided a relatively good regression coefficient value (r^2^) (see Table 5). The fitting provided an n value slightly higher than 1. When fitting the results to the Korsmeyer–Peppas model, an n > 1 indicated a clearly non-Fickian permeation process, dominated by significant swelling, erosion, or hydration effects. This behavior was consistent with the PEO nanofibers undergoing considerable swelling and gelling during the release of the caffeine.

## 4. Conclusions

This study demonstrated the feasibility of using electrospinning technology for generating innovative bilayer platforms for the transdermal delivery of drugs, using caffeine as a model compound. As anticipated, the use of specific enhancers compatible with both the drug and skin composition, was confirmed to enhance transdermal drug delivery. The results indicated that patches containing both polyethylene glycol (PEG) and oleic acid (OA) as enhancers exhibited the highest synergistic effect on the caffeine permeability when it was tested with an artificial membrane. This patch was further assessed in transdermal assays using ex vivo human SC specimens from two very different donors as the membranes, suggesting higher variability in the permeation with longer testing times, which was ascribed to the small size of the patch tested in the Franz cell, i.e., 1 cm^2^, and potential tissue deterioration over longer testing periods. The best-performing platform achieved average drug permeation rates of 0.73 mg/cm^2^ across the synthetic membrane after 24 h.

## Figures and Tables

**Table 1:** Composition of PEO/caffeine solutions.

Sample ID	Ratio of Polymer/API/Enhancer (*w/w*)	Solvents and Ratio (*w/w*)
PEO_CAF	80/20	Chloroform/MeOH (80:20)
PEO_CAF_OA	70/20/10	
PEO_CAF_PEG	64/20/16	
PEO_CAF_PG_OA	56/20/14/10	
PEO_CAF_PEG_OA	56/20/14/10	
PEO_CAF_PEG_EUC	60/20/15/5	

**Table 2:** Electrospinning parameters for the PEO-containing caffeine solutions with different enhancers and the backing layer (BL).

Sample ID	Flow Rate (mL/h)	Voltage, V+/V− (kV)	Needle-to-Collector Distance (cm)
PEO_CAF	10	20/−5	20
PEO_CAF_OA	10	20/−10	20
PEO_CAF_PEG	10	20/−10	20
PEO_CAF_PG_OA	10	20/−10	20
PEO_CAF_PEG_OA	10	20/−10	20
PEO_CAF_PEG_EUC	10	20/−10	20
BL (PCL)	20	15/−2	15

**Table 3:** Fiber size in PEO/caffeine active layers with different enhancers and presence of caffeine at fiber surface.

Sample ID	Fiber Size (µm)	Caffeine at Fiber Surface
PEO_CAF	2.1 ± 0.5	Yes
PEO_CAF_OA	2.8 ± 0.4	Yes
PEO_CAF_PEG	2.3 ± 0.4	Yes
PEO_CAF_PG_OA	2.2 ± 0.4	Yes
PEO_CAF_PEG_OA	1.1 ± 0.2	No
PEO_CAF_PEG_EUC	1.7 ± 0.2	No

**Table 4:** Initial caffeine content in the multilayer PCL + PEO/caffeine/enhancer patch and the corresponding permeated caffeine after 24 h.

Sample ID	Initial Caffeine Content (mg/cm^2^)	Permeated Caffeine (mg/cm^2^)
PEO_CAF	1.33	0.15 ± 0.01
PEO_CAF_OA	1.87	0.17 ± 0.02
PEO_CAF_PEG	1.85	0.31 ± 0.08
PEO_CAF_PG_OA	1.95	0.17 ± 0.04
PEO_CAF_PEG_OA	2.02	0.73 ± 0.02
PEO_CAF_PEG_EUC	1.69	0.41 ± 0.34

**Table 5:** Model parameters of the human SC caffeine permeation profiles of the PCL + PEO_CAF_PEG_OA multilayer patch obtained from the application of the Korsmeyer–Peppas model.

ID	Korsmeyer–Peppas		
	K	n	r^2^
PCL + PEO_CAF_PEG_OA multilayer patch	0.63	1.09	0.96

## Data Availability

Available upon reasonable request.
